# Antiretroviral Therapy (ART) Adherence and Prenatal Alcohol Use among Women Who Are Pregnant with HIV in South Africa

**DOI:** 10.3390/ijerph18147446

**Published:** 2021-07-13

**Authors:** Yukiko Washio, Felicia A. Browne, Jacqueline Ndirangu, Tracy L. Kline, Wendee M. Wechsberg

**Affiliations:** 1 Substance Use, Gender and Applied Research, RTI International, Research Triangle Park, NC 27709, USA; fbrowne@rti.org (F.A.B.); jndirangu@rti.org (J.N.); tkline@rti.org (T.L.K.); wmw@rti.org (W.M.W.); 2 Department of Obstetrics, Gynecology and Reproductive Sciences, Temple University Lewis Katz School of Medicine, Philadelphia, PA 19122, USA; 3 Gillings School of Global Public Health, University of North Carolina (UNC), Chapel Hill, NC 27599, USA; 4 Psychiatry and Behavioral Sciences, Duke University School of Medicine, Durham, NC 27708, USA; 5 Psychology, North Carolina State University, Raleigh, NC 27606, USA

**Keywords:** ART adherence, prenatal alcohol use, fetal alcohol spectrum disorders, brief behavioral intervention, South Africa

## Abstract

This brief report emphasizes the need to focus on women with HIV who are pregnant who use alcohol or other drugs. A recently completed implementation science study tested a gender-focused behavioral intervention, the Women’s Health CoOp (WHC), to improve antiretroviral therapy (ART) adherence and reduce alcohol use among women with HIV. The study identified 33 participants who had a positive pregnancy test result at the baseline assessment, of whom five participants remained pregnant during the 6-month duration of the study. Of the 33 pregnant participants at the baseline assessment, 55% reported past-month alcohol use, with 27% reporting a history of physical abuse and 12% reporting a history of sexual abuse. The five women who remained pregnant at 6 months showed improved ART adherence and reduced prenatal alcohol use. The gender-focused WHC intervention shows promise as a cost-effective, sustainable, behavioral intervention to address these intersecting syndemic issues. Future research should focus on identifying the needs of women with HIV who are pregnant who use alcohol or other drugs and developing tailored evidence-based behavioral interventions such as the WHC for preventing FASD in addition to improving ART adherence in this key population of women and reducing the economic burden on society.

## 1. Introduction

South Africa has the largest HIV epidemic globally, with 7.1 million people with HIV and over 20% prevalence among the general population [1]. In response, South Africa has the largest HIV treatment program in the world [1]. HIV-related issues, including HIV acquisition and medication adherence, are often associated with psychosocial factors such as intimate partner violence (IPV) and substance use. The confluence of substance use, violence, and HIV has been labeled the SAVA (substance abuse, violence, and HIV/AIDS) syndemic [2]. Women of historically underserved racial/ethnic groups are represented disproportionately in this syndemic, demonstrating how these intersectional issues affect women and that each issue cannot be addressed as a single solution [2].

The syndemic of prenatal alcohol use, IPV, and HIV is particularly common in South Africa [3], with the highest reported rates of fetal alcohol spectrum disorders (FASD) [4]. Prenatal exposure to alcohol is associated with birth and neonatal complications such as miscarriage, a low birth weight, stillbirth, and a range of lifelong disorders, including FASD [5].

In resource-limited settings, it is recommended that women with HIV adhere to antiretroviral therapy (ART) and breastfeed their children up to a year after birth [6]. However, ART initiation and adherence among women who are pregnant with HIV are significantly associated with support from their male partners, peers, and families and marital status, cigarette smoking, and alcohol use [7].

The evidence suggests the importance of promoting ART adherence among women with HIV who are pregnant in South Africa and addressing other important syndemic issues such as prenatal alcohol use and IPV exposure. South Africa has well documented both FASD and HIV separately but not as intersecting phenomena. Additionally, a recent review was not able to identify many interventions targeting alcohol use among women with HIV, including women who were pregnant [8]. This brief report focuses on the characteristics and outcomes of pregnant participants who were part of a recently completed implementation science trial in South Africa to emphasize the importance of tailoring an evidence-based intervention for women with HIV and who report prenatal alcohol use, with the aim of preventing FASD in addition to improving ART adherence and reducing the overall economic burden on society. 

## 2. Materials and Methods 

A recently completed implementation science trial in South Africa [9,10,11] to improve ART adherence and reduce alcohol use among women with HIV enrolled a total of 480 women recruited via HIV testing and counseling (HTC) clinics and substance use rehabilitation centers in economically disadvantaged communities in Cape Town, South Africa. A modified stepped-wedge design was applied in which each HTC clinic was paired with a substance use rehabilitation center and each pair was randomized by computer to one of four implementation cycles [10]. This brief report will report on only a subset of 33 participants who had a positive pregnancy test result at the baseline assessment. All pregnant participants were provided with an active referral for antenatal care. Of these 33 participants, five women remained pregnant during the 6-month duration of the study, indicating the importance of addressing prenatal alcohol use for FASD prevention and focusing on ART adherence among women with HIV. We did not collect information of what happened to pregnancies that ended during the study period. 

Study participants had to (1) be between the ages of 18 and 45 years, (2) report the use of at least one drug or alcohol at least weekly during the previous 3 months, (3) report condomless sex with a male partner in the past 6 months, (4) have a positive verifiable HIV test result, (5) report the intention to remain in the area for at least the next 6 months, (6) provide contact information, and (7) be willing to participate in alcohol and other drug screening. Data collection included the baseline and 6-month follow-up assessments on the intervention outcomes. Assessment measures included socioeconomic status, sex risk behaviors, alcohol and other drug use, substance use treatment readiness, ART and barriers to use, and other self-reported clinical outcomes such as tuberculosis and symptoms of sexually transmitted infections. In addition to self-reported alcohol and other drug use, urine screening was conducted for recent drug use and a breathalyzer test for alcohol use. A clinic-issued document such as an antiretroviral (ARV) card or ARV medication with identifiable data linking to the participant was required to verify an HIV-positive status. For participants who did not have this documentation, consent was requested to access their hospital records to check their HIV status. 

The trial implemented the Women’s Health CoOp (WHC) intervention, which addresses the intersecting risks of substance use, sexual behaviors, and violence and victimization with the primary goal of increasing skills and knowledge to reduce substance use and other HIV-related risks among women [10]. The WHC comprises two sessions in the form of group workshops or individual sessions held approximately one week apart, with each session lasting about an hour or longer depending on the discussion and translations. In addition to education, the sessions consist of skill development through role-play and rehearsal such as steps for proper male and female condom use, sexual negotiation, and violence prevention strategies. At the end of the second session, participants have an opportunity to set personal goals to reduce their specific risks. Intervention staff also provide referrals to treatment and other available services.

## 3. Results

The participant characteristics of the 33 participants who had a positive pregnancy test result at the baseline assessment are summarized in Table 1. The average participant age was older than 30 years, with most having fewer than 12 years of education and having a main partner. Besides the 55% who reported past-month alcohol use, 9 out of 33 participants provided alcohol-positive breathalyzer samples at the baseline assessment. Urine screening at the baseline assessment showed that methamphetamine was the most prevalent substance used among these pregnant participants. Additionally, past physical abuse was a more prevalent form of abuse compared with sexual abuse. 

Among the five participants who remained pregnant at the 6-month follow-up, past-month ART adherence was 80% at the baseline assessment and 100% at the follow-up assessment. Self-reported 85% or a greater ART adherence was 60% for participants at the baseline assessment and 80% at the follow-up assessment. Alcohol use frequency and quantity measures classified whether a participant was at risk for an alcohol use disorder. All five participants were at risk for an alcohol use disorder at the baseline assessment; however, only two participants were at risk at the follow-up assessment and no positive breathalyzer test results were indicated at the follow-up. Of the five participants, four had completed both WHC intervention sessions. One participant reported physical abuse exposure in the past 6 months at the baseline assessment but no physical abuse at the follow-up assessment. The average number of days of alcohol use in the past month was 12 ± 4.3 at the baseline assessment and 4.8 ± 7.2 at the follow-up assessment. At the baseline assessment, prenatal alcohol use and IPV exposure were prevalent among pregnant participants with HIV. Future research should continue to test with a much larger sample size the effect of the WHC intervention tailored to address prenatal alcohol use and ART adherence to understand its impact on both public health issues of FASD and HIV/AIDS among women with HIV who are pregnant.

Although the study did not collect ARV medication information from participants, ART initiation was verified with clinic records. As recommended by the South African national guidelines [12] for women with HIV who are pregnant, most participants were taking a single, fixed-dose combination (FDC) regimen containing tenofovir disoproxil fumarate (TDF), lamivudine/emtricitabine (3TC/FTC), and efavirenz (EFV) if they did not have contraindications to the FDC component drugs. 

The implementation of the WHC program continued a year after operations ended in several of the facilities where components of or the full intervention were used. The content of the program can be modified to make it relevant for various populations and adapted to different settings within Africa and globally. While working on the dissemination of the findings, the local government is planning to continue with the WHC intervention. Challenges encountered during the implementation study included the management of staff schedules, other staff demands, clinic space issues, staff attitudes toward patients such as stigma, and analyses of implementation study data because of changes in samples of patients and staff members [11].

As noted earlier, the WHC addresses the syndemic issues of substance use, sexual behaviors and violence, which are relevant to HIV risk reduction and treatment adherence. Programs and interventions that integrate treatment for HIV and other related issues such as the WHC in usual care settings can be cost-effective. For women who are pregnant with HIV in South Africa who also are at risk of prenatal alcohol use, the economic burdens for having children with FASD and/or HIV should also be taken into consideration.

In the US, the annual economic cost of FASD to the healthcare system is more than USD 6 billion and the cost of care for an individual with FASD can be up to USD 2 million [13]. The care costs for individuals with severe FASD can be three times more than for those with mild FASD [14]. The healthcare costs for children with FASD are nine times higher than for children without FASD (USD 20,017 compared with USD 2217 in 2013 dollars) [15]. The healthcare costs to treat children with FASD aged 0 to 2 are the highest because of neonatal intensive care unit admissions to treat low birth weight babies with FASD and the surgical correction of FASD-related birth defects [14]. Unit costs for treating individuals with HIV ranges from approximately USD 1900 to USD 5000 in high-income countries such as the US [16]. Additionally, if women do not adhere to ART and mother-to-child transmission occurs at birth or via breastfeeding, additional pediatric ART costs for a 5-year-old or younger is estimated to be approximately USD 30 per month [17]. Future intervention and implementation research should include components to track the cost outcomes and conduct cost-effectiveness analyses to weigh the cost of tested treatment interventions against the economic burden of the existing treatment.

## 4. Conclusions

This brief report addresses the importance of focusing on pregnant participants living with HIV in South Africa who use alcohol or other drugs because they are at risk of having children with FASD and IPV exposure, which could potentially adversely impact ART adherence. A gender-focused intervention such as the WHC has shown promise as a cost-effective, sustainable, behavioral intervention to address these intersecting syndemic issues. Future research should focus on identifying the needs of women who are pregnant with HIV who use alcohol or other drugs and developing tailored interventions based on an evidence-based behavioral intervention such as the WHC for preventing FASD in addition to improving ART adherence in this key population of women and, ultimately, reducing the economic burden on society.

## Figures and Tables

**Table 1:** Participant baseline characteristics of women who were pregnant at the baseline assessment (N = 33).

Variables	N(%)/M(SD)
Education (9th grade plus)	27 (82%)
Home—Metal Walls	17 (52%)
Brick Walls	8 (24%)
Cement Walls	8 (24%)
Home—Inside Running Water	24 (73%)
Have Main Partner	30 (91%)
Age	32 ± 5.7
Past Month Drinking Self-Report	18 (55%)
Methamphetamine Urine Screening	2 (6%)
Opiates Urine Screening	0 (0%)
Marijuana Urine Screening	1 (3%)
Mandrax Urine Screening	1 (3%)
Past 6 Months Physical Abuse	8 (24%)
Past Physical Abuse (Longer than 6 months ago)	9 (27%)
Past Sexual Abuse (Longer than 6 Months ago)	4 (12%)

## Data Availability

Data will be deposited to NIAAA data archive.
